# Association between PD-L1 expression and driver gene status in non-small-cell lung cancer: a meta-analysis

**DOI:** 10.18632/oncotarget.23969

**Published:** 2018-01-05

**Authors:** Bo Lan, Chengxi Ma, Chengyan Zhang, Shoujie Chai, Pingli Wang, Liren Ding, Kai Wang

**Affiliations:** ^1^ Department of Respiratory Medicine, Second Affiliated Hospital, Zhejiang University School of Medicine, Hangzhou, China; ^2^ Department of Oncology, Ningbo First Hospital, Ningbo, China

**Keywords:** PD-L1, driver gene, NSCLC

## Abstract

**Objective:**

To assess the association between PD-L1 expression and driver gene mutations in patients with non-small-cell lung cancer (NSCLC).

**Method:**

We performed a meta-analysis of 26 studies (7541 patients) which were published from 2015 to 2017. Pooled odds ratios (ORs) with 95% confidence intervals (CI) were calculated to describe the correlation. Subgroup analysis was performed based on population characteristics, types of PD-L1 antibodies and quality of individual studies.

**Results:**

A lower frequency of PD-L1 positivity was observed in NSCLCs harboring EGFR mutation (OR: 0.64, 95% CI, 0.45–0.91, *p* = 0.014). A negative correlation was also found at 1% (OR: 0.35, 95% CI, 0.22–0.55, *p* = 0.000) and 50% (OR: 0.33, 95% CI, 0.14–0.81, *p* = 0.015) cutoff for PD-L1 positive, elderly age group (OR: 0.56, 95% CI, 0.35–0.89, *p* = 0.013), female dominant group (OR: 0.55, 95% CI, 0.29–0.94, *p* = 0.030) and smoker dominant group (OR: 0.52, 95% CI, 0.29–0.96, *p* = 0.035). No significant differences in PD-L1 expression were observed among patients with different ALK, BRAF, HER2, PIK3CA status and MET expression level. Higher level of PD-L1 was found in tumors with KRAS mutation (OR: 1.45, 95% CI, 1.18–1.80, *p* = 0.001). PD-L1 expression level was not significantly different between triple (EGFR/ALK/KRAS) wild type NSCLCs and those with EGFR/ALK/KRAS mutation.

**Conclusions:**

PD-L1 expression in EGFR mutated NSCLCs were lower than those in EGFR wild type NSCLCs, while tumors with KRAS mutation showed higher levels of PD-L1.

## INTRODUCTION

Lung cancer is the most common cancer and the leading cause of cancer-related mortality around the world. In 2015, a total of 733,300 patients (509,300 men and 224,000 women) were diagnosed with lung cancer in China [1]. Non-small-cell lung cancer (NSCLC) and small cell lung cancer (SCLC) are two main types of lung cancers; of these, NSCLC accounts for 80–85% of all lung cancers [2]. Unfortunately, about 53% patients of NSCLC are diagnosed at an advanced stage (III b-IV) and, therefore, have a poor prognosis [3].

Programmed cell death 1 ligand 1 (PD-L1; also referred to as B7-H1), which belongs to B7 family, is an inhibitory cell surface molecule. PD-L1 expressed on NSCLC cells is shown to inhibit T cell proliferation and activation by combining with programmed cell death 1 (PD-1) receptor. Blockade of PD-1/PD-L1 represents a novel target for immunotherapy [4]. In a recent clinical trial, Keynote-024, [5]. progression free survival (PFS) in the pembrolizumab group was 4.3 months longer than that in the chemotherapy group (10.3 vs. 6.0 months; hazard ratio [HR]: 0.50, 95% confidence interval [CI], 0.37–0.68). Based on the promising results, anti-PD-1 /PD-L1 agents, nivolumab, pembrolizumab and atezolizumab were approved by FDA for treatment of advanced NSCLCs in the past few years [6–9].

Driver gene alternations including epidermal growth factor receptor (EGFR), anaplastic lymphoma kinase (ALK), and Kirsten rat sarcoma viral oncogene homolog (KRAS) have been verified in a large proportion of NSCLCs. For example, 45% of never smokers and 7% of patients with tobacco-associated lung cancer harbor EGFR mutations. At least 30% of East Asian patients with NSCLC have EGFR mutation as compared to <10% of Caucasian patients [10–12]. *ALK* rearrangements containing exon 20 coding for the tyrosine domain occur in 3.0% to 11.5% of patients with NSCLC [13]. KRAS mutations have been reported in 15–30% of patients with NSCLC, and approximately 97% of mutations are point mutations located in codons 12 or 13 of exon [14].

Gainor *et al.* reported an objective response rate (ORR) of 3.6% in EGFR mutation or ALK rearrangement cohort treated with pembrolizumab, while the ORR in EGFR/ALK wild type cohort was 23.3%. Similarly, in Checkmate 057 and Keynote-010, neither nivolumab nor pembrolizumab showed an obvious superiority over docetaxel with respect to overall survival (OS) in the EGFR mutation cohorts [15]. In contrast to EGFR/ALK, KRAS mutation was associated with better outcomes (versus docetaxel) with respect to OS in Checkmate 057 (HR: 0.52, 95% CI, 0.29–0.95). PD-L1 expression in tumor issues is considered as a predictor of immunotherapy outcomes, and its’ relationship with driver gene status has attracted increasing attention in recent years.

Results of preclinical studies indicate a positive correlation between EGFR, ALK and KRAS mutations and higher PD-L1 levels, although the underlying mechanisms are not clear. EGFR mutation may upregulate PD-L1 expression through PI3K, MAPK, STAT3 and NF-κB signaling pathway [16, 17]. Similarly, PI3K, MAPK, STAT3 and HIF-1α signaling pathways may be involved in upregulation of PD-L1 expression in ALK mutated cell lines [18, 19]. Treatment with tyrosine kinase inhibitors (TKIs), gefitinib and crizotinib was shown to decrease PD-L1 expression levels *in vitro* [17]. Co-mutation of STK11/LKB1 and KRAS was shown to downregulate PD-L1 expression level in NSCLC cell lines; in contrast, co-mutation of tp53 and KRAS was shown an upregulating effect [20, 21]. However, the association between PD-L1 expression and driver gene status in clinical tumor specmens is not well characterized [22]. This meta-analysis was aimed at further evaluation of the association between PD-L1 expression and driver gene mutation in order to explain variable outcomes of immunotherapy in patients with NSCLCs harboring various driver gene status.

## RESULTS

### Search result and selection of studies

**Figure 1:**
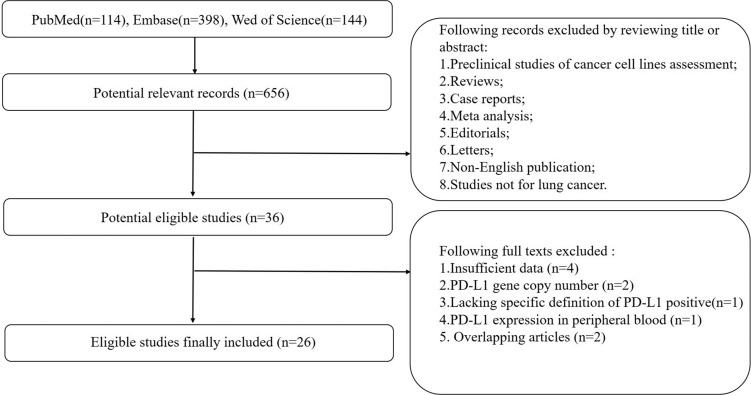
Flow diagram showing selection of studies

The MOOSE guidelines were followed for this meta-analysis of observational studies [23]. Flow diagram showing study selection procedure is presented (Figure 1). Twenty-five publications and one meeting abstract (2015–2017) with a combined study population of 7541 patients were finally included. Due to the higher prevalence of driver gene (especially EGFR and ALK) in East-Asian population, 19 studies were conducted on East-Asian population, while 8 studies were conducted on Caucasian or American population. Quality assessment using STROBE checklist showed a 57.5% median total score (Supplementary Table 1); one study was classified as “high-quality”; one study was classified as “low-quality”; and the remaining studies were classified as “moderate-quality” (Table 1). The percentage of tumor cells staining positive for PD-L1 was calculated for each specimen. The optimal cutoff level is still controversial; 5% tumor cells staining positive is the most common cutoff level used in clinical trials. Baseline population characteristics including median age, gender, tumor stage, histological types and smoking status are summarized in Table 1. For clinical specimens, 1598 (32.67%), 99 (3.25%) and 628 (19.83%) were reported harboring EGFR, ALK and KRAS mutations, respectively (Supplementary Table 2).

**Table 1:** Basic characteristics of studies included in the meta-analysis

First author	Year of publication		No of patients	Ethnicity	Median age(ys)	Gender	Smoking status	Stage	Histology	Antibody	Cutoff	Quality
Zhang [33]	2014		143	East-Asian	<60	Male (41%) Female (59%)	Smoker (34%) Non-Smoker (66%)	I (46%) II–III (54%)	ADC^a^ (100%)	PoAb^e^	40%	M1
Yang [34]	2014		163	East-Asian	≥60	Male (33%) Female (67%)	Smoker (19%) Non-Smoker (81%)	I (100%)	ADC (100%)	PoAb	5%	M
Incecoo [35]	2014		123	Other	≥60	Male (54%) Female (46%)	Smoker (60%) Non-Smoker (30%)	NRg	SCC^b^ (66%) ADC (18%) Other (15%)	PoAb	5%	M
Cooper [36]	2015		678	Other	≥60	Male (70%) Female (30%)	NR	I (50%) II–III (50%)	SCC (41%) ADC (40%) Other (19%)	McAb^f^	50%	M
Schmidt [37]	2015		321	Other	Other	Male (78%) Female (22%)	Smoker (81%) Non-Smoker (20%)	I (58%), II (26%) III (16%)	SCC (46%) ADC (39%) Other (15%)	McAb	5%	M
Kim [38]	2015		331	East-Asian	≥60	Male (96%) Female (4%)	Smoker (95%) Non-Smoker (5%)	I (40%) II (36%) III (24%)	SCC (100%)	McAb	10%	M
Chang [39]	2015		66	East-Asian	<60	Male (38%) Female (62%)	Smoker (12%) Non-Smoker (88%)	I (32%) II (24%) III–IV (21%)	LELC^c^ (100%)	PoAb	5%	M
Koh [40]	2015		497	East-Asian	≥60	Male (46%) Female (54%)	Smoker (40%) Non-Smoker (60%)	NR	ADC (100%)	McAb	5%	M
Tang [41]	2015		170	East-Asian	<60	Male (55%) Female (45%)	Smoker (34%) Non-Smoker (66%)	III B (5%) IV (95%)	ADC (85%) Other (15%)	McAb	5%	M
Omori [42]	2015		95	East-Asian	≥60	Male (63%) Female (37%)	Smoker (74%) Non-Smoker (26%)	I (32%) II (24%) III (21%)	SCC (16%) ADC (78%) Other (6%)	McAb	1%	M
Yang2 [43]	2016		105	East-Asian	≥60	Male (85%) Female (15%)	Smoker (75%) Non-Smoker (15%)	I (100%)	SCC (100%)	PoAb	5%	M
Andreas [44]	2016		436	Other	≥60	Male (54%) Female (46%)	Smoker (89%) Non-Smoker (10%)	I (47%) II (13%) III (33%) IV (7%)	ADC (100%)	McAb	1%	M
Ameratunga [45]		2016	527	Other	≥60	Male (69%) Female (31%)	Smoker (90%) Non-Smoker (7%)	NR	SCC (34%) ADC (55%) Other (11%)	McAb	5% 50%	M
Song [46]	2016		385	East-Asian	<60	Male (51%) Female (49%)	Smoker (39%) Non-Smoker (61%)	I (31%) II (21%) III (48%)	ADC (100%)	McAb	5%	M
Inamura [47]	2016		268	East-Asian	≥60	Male (53%) Female (47%)	Smoker (58%) Non-Smoker (42%)	I (56%) II–III (44%)	ADC (100%)	McAb	1%, 5%	M
Jia [48]	2016		55	East-Asian	≥60	Male (53%) Female (47%)	Smoker (31%) Non-Smoker (69%)	NR	SDPLC^d^ (100%)	McAb	10%	M
Inoue [49]	2016		654	East-Asian	≥60	Male (68%) Female (32%)	Smoker 68%) Non-Smoker (30%)	I (64%) II (17%) III (19%)	SCC (27%) ADC (66%) Other (11%)	McAb	5%	M
Ji [50]	2016		100	East-Asian	≥60	Male (51%) Female (49%)	Smoker (26%) Non-Smoker (74%)	I 42%) II (27%) III (31%)	ADC (100%)	PoAb	5%	M
Huynh [51]	2016		261	Other	≥60	Male (35%) Female (65%)	Smoker (78%) Non-Smoker (22%)	I (77%) II (13%) III (8%) IV (2%)	ADC (100%)	McAb	5%	M
Mori [52]	2016		296	East-Asian	≥60	Male (50%) Female (50%)	Smoker (52%) Non-Smoker (48%)	NR	ADC (100%)	McAb	Score 50	M
Takada [53]	2016		417	East-Asian	≥60	Male (49%) Female (51%)	Smoker (48%) Non-Smoker (52%)	I (73%) II–III (27%)	ADC (100%)	McAb	1%5%	M
Dong [54]	2017		13	East-Asian	≥60	Male (92%) Female (8%)	Smoker (52%) Non-Smoker (48%)	III (23%) IV (77%)	SCC (23%) ADC (69%) Other (8%)	McAb	5%, 50%	M
Rangachari [55]	2017		71	Other	Other	Male (45%) Female (55%)	Smoker (68%) Non-Smoker (32%)	I–III (25%) IV (75%)	ADC (100%)	McAb	50%	L^2^
Tsao [56]	2017		982	Other	Other	Male (73%) Female (27%)	NR	I (46%) II (37%) III (8%) IV (2%)	SCC (45%) ADC (41%) Other (14%)	McAb	1%, 25% 50%	H^3^
Chen [57]	2017		65	East-Asian	≥60	Male (57%) Female (43%)	Smoker (52%) Non-Smoker (48%)	I + II (51%) III (49%)	ADC (100%)	NR	5%	M
Cho [58]	2017		319	East-Asian	≥60	Male (39%) Female (61%)	Smoker (36%) Non-Smoker (64%)	I (63%) II (13%) III (19%) IV (5%)	ADC (97%) Other (3%)	McAb	1%, 50%	M

Note: a: “ADC” represents adenocarcinoma; b: “SCC” represents squamous-cell carcinoma; c: “LELC” represents lymphoepithelioma-like carcinoma; d: “SDPLC” represents synchronous double primary lung cancer, and 2 cases of SCLC were excluded; e: “PoAb” represents “polyclonal antibody”; f: “McAb” represents “monoclonal antibody”; g: “NR” represents “not reported”; 1: “M” represents moderate quality; 2: “L” represents low quality; 3: “H” represents high quality.

### EGFR mutation versus EGFR wild type

**Figure 2:**
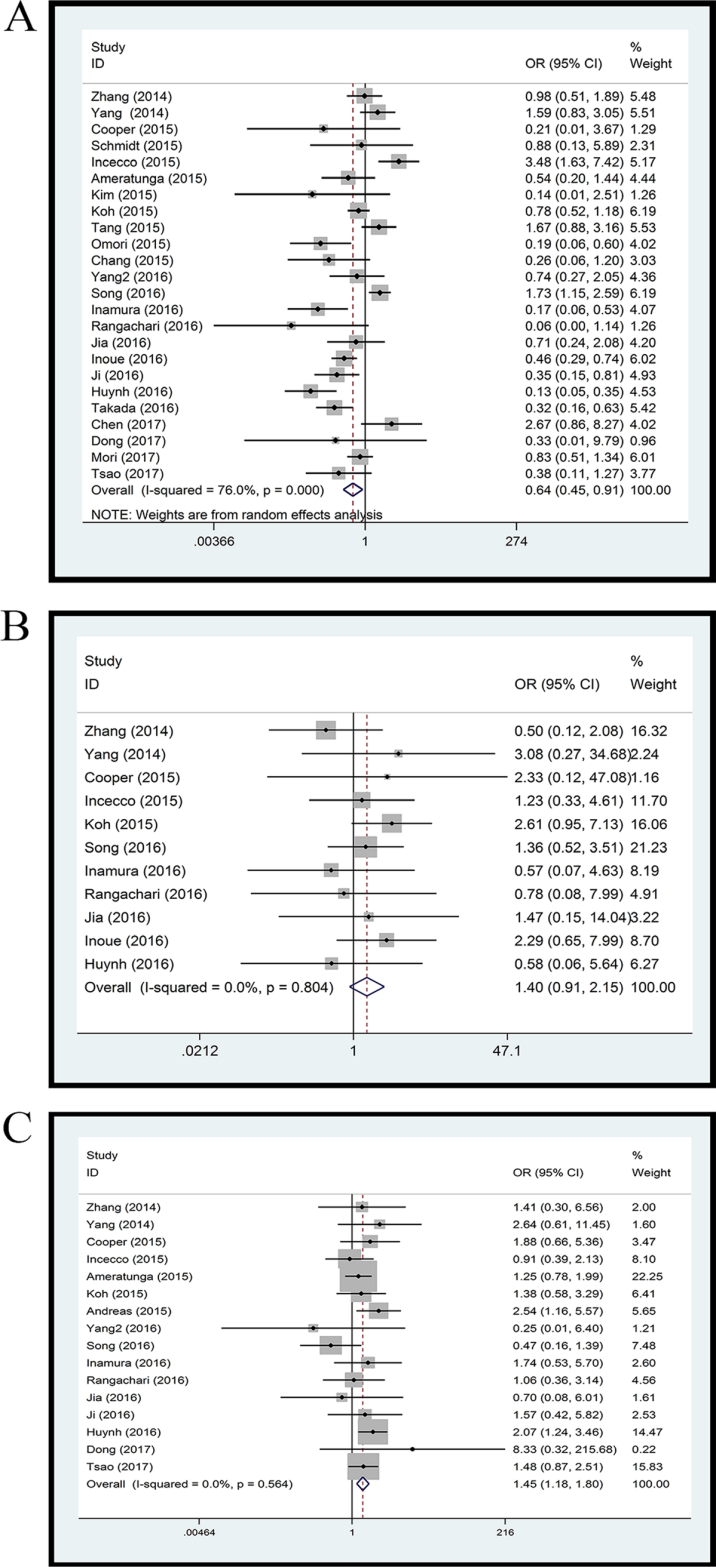
Forest plot (**A**) EGFR mutation versus EGFR wild type; (**B**) ALK mutation versus ALK wild type; (**C**) KRAS mutation versus KRAS wild type.

**Figure 3:**
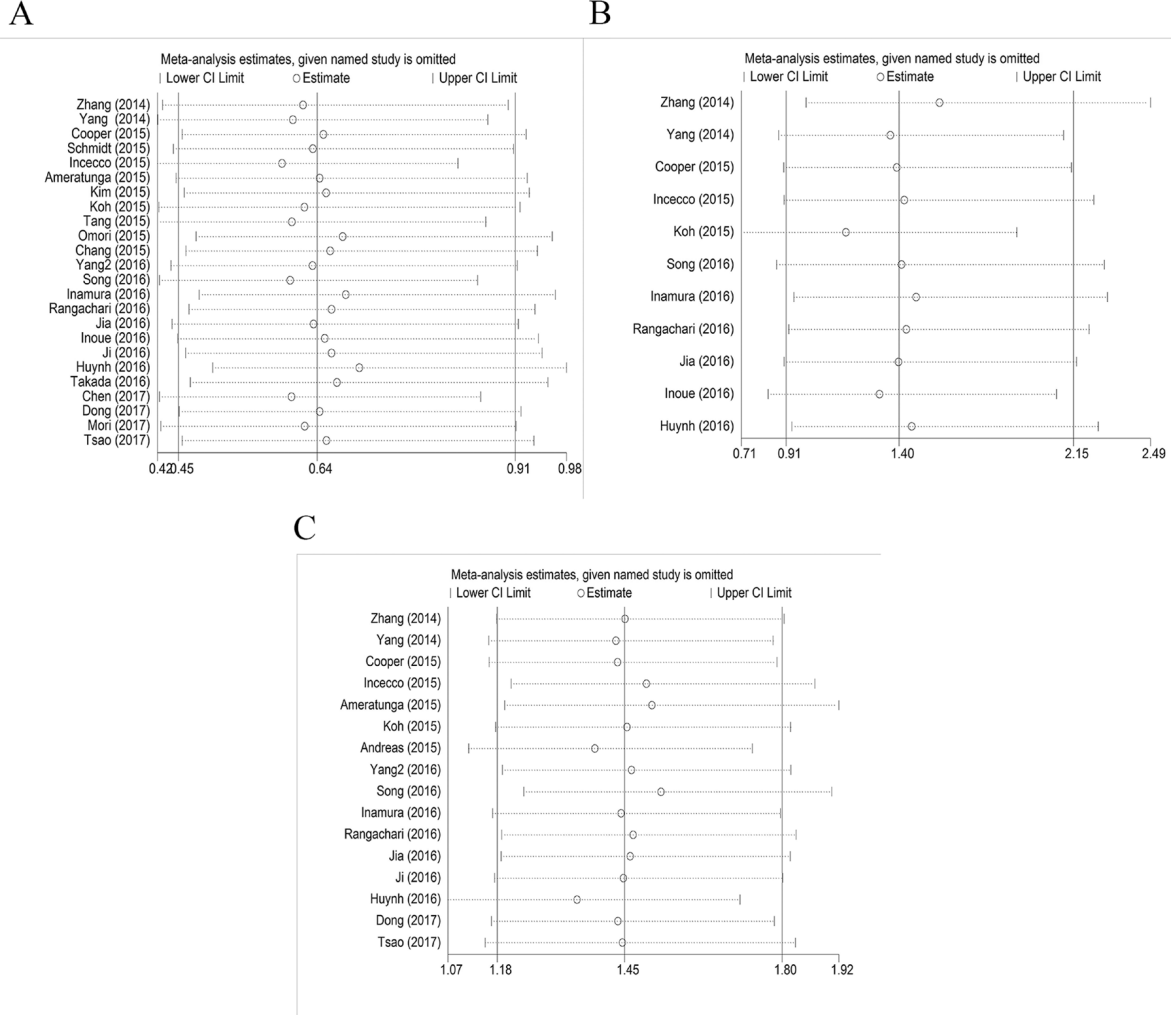
Sensitivity analysis (**A**) EGFR mutation versus EGFR wild type; (**B**) ALK mutation versus ALK wild type; (**C**) KRAS mutation versus KRAS wild type.

**Figure 4:**
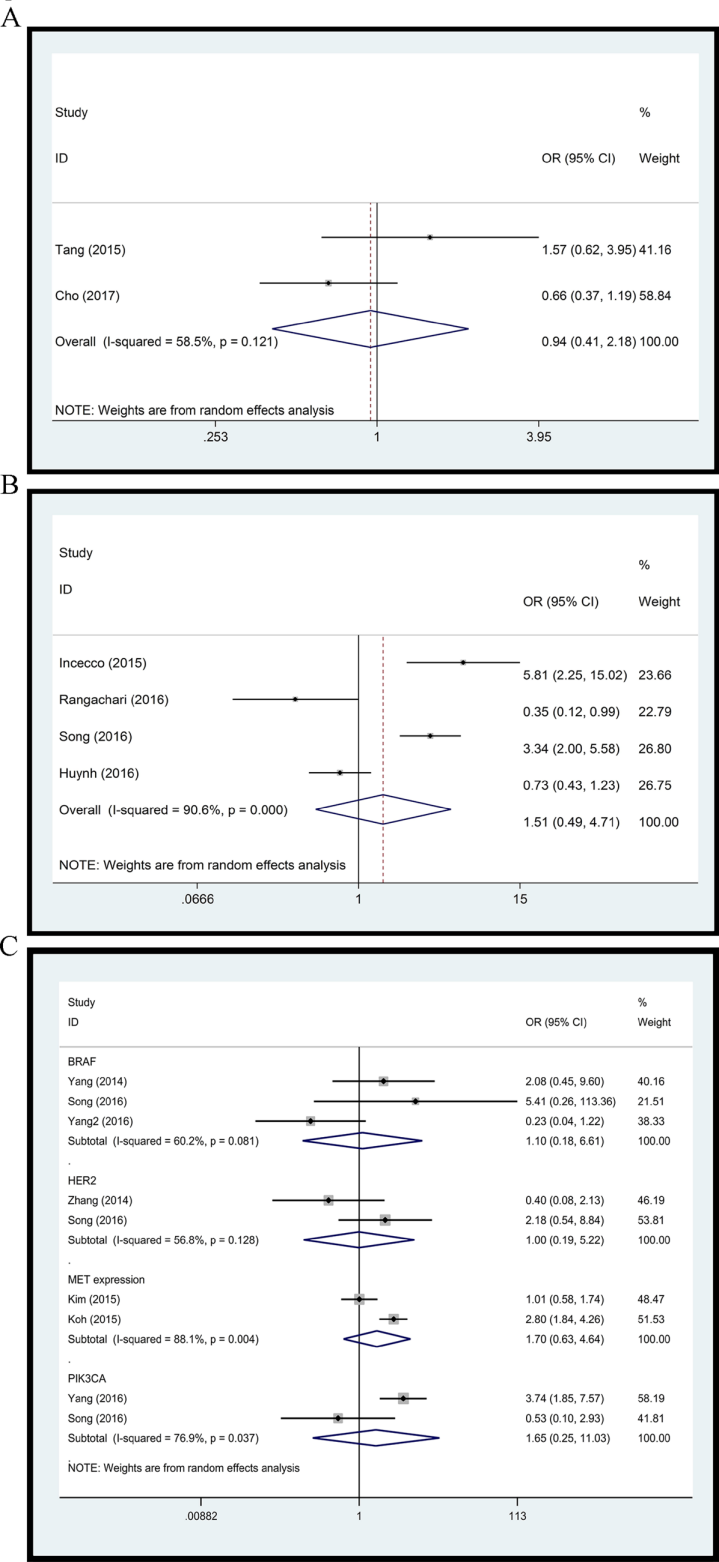
Forest plot (**A**) L858R versus Del19; (**B**) EGFR/ALK/KRAS mutation versus triple wild type; (**C**) BRAF, HER2, PIK3CA status and MET expression.

**Figure 5:**
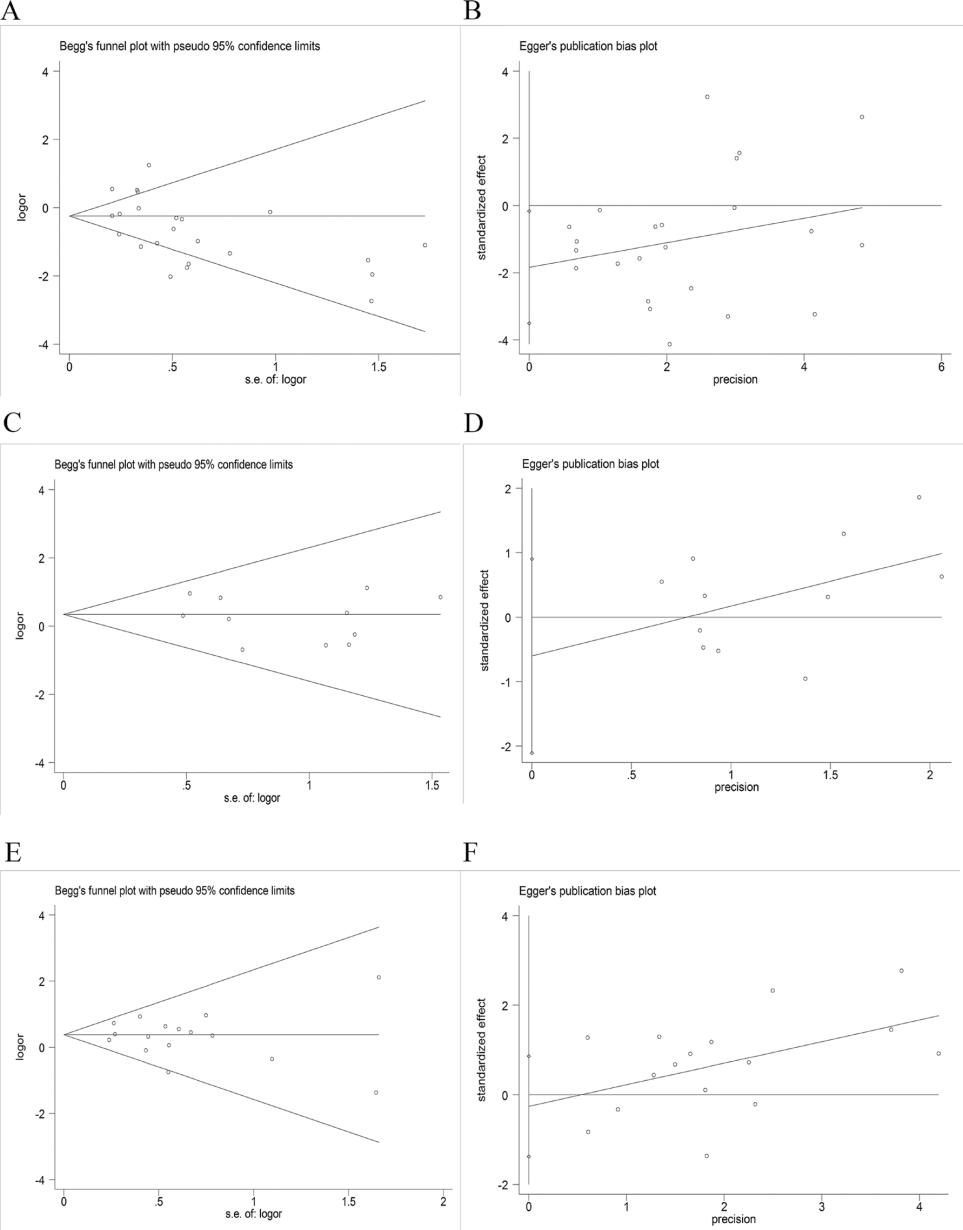
Assessment of publication bias (**A** and **B**) Begg’s test and Egger’s test of EGFR mutation versus EGFR wild type; (**C** and **D**) Begg’s test and Egger’s test of ALK mutation versus ALK wild type; (**E** and **F**) Begg’s test and Egger’s test of KRAS mutation versus KRAS wild type.

Twenty-four studies with 4891 clinical specimens were included. A high level of heterogeneity was observed among the included studies (*Q* = 93.67, *I*^*2*^ *=* 75.4%, *p =* 0.000). Using a random effect model, we found a statistically significant negative correlation between PD-L1 expression and EGFR mutation in NSCLCs (OR = 0.64, 95% CI = 0.45–0.91, *p =* 0.014) (Figure 2A). On sensitivity analysis, respective pooled ORs remained significantly stable after sequential elimination of individual studies (Figure 3A). In order to detect the origin of heterogeneity and to assess the influence of different population characteristics, we set up a series of subgroups according to histological type, cutoff level for PD-L1 positivity, population characteristics (median age, ethnicity, stage, gender, smoking status), types of primary antibodies and study quality. Meta regression was performed to examine the influence of each parameter on heterogeneity. However, neither subgroup analysis nor meta regression could identify any parameter which was responsible for high heterogeneity (Table 2). We also explored potential differences of PD-L1 expression among diverse EGFR mutation status [exon 19 deletion (Del19) and codon 858 mutation in exon 21 (L858R)]; however, no evidence was found to validate our hypothesis (Figure 4A). Furthermore, pooled data showed a significant negative correlation between PD-L1 and EGFR mutation status when the cutoff level for PD-L1 positivity was 1% and 50%. Similar results were found in East-Asian, elder/female/smoker dominant groups (Table 2). In terms of potential publication bias, statistical results of Begg’s test and Egger’s test (Begg’s *p =* 0.126 and Egger’s *p =* 0.033) were inconsistent (Figure 5A and 5B). However, no trimming was performed during the trim and fill procedure, which indicated that dissymmetry of funnel plots may have resulted from other parameters rather than publication bias.

**Table 2:** Association between PD-L1 expression and driver gene status among different clinicopathological conditions

Driver gene	Subgroup	Studies	OR	95% CI	*p*	Heterogeneity test			
						*Q*	*I*^2^	*p*^1^	*p*^2^
EGFR									
	Median age(years)								
	≥60	16	0.56	0.35–0.89	0.013	64.05	76.6%	0.000	0.109
	<60	4	1.18	0.70–2.00	0.526	6.84	56.1%	0.077	
	Gender								
	Male > Female	16	0.68	0.41–1.12	0.129	62.45	76.0%	0.000	0.720
	Male ≤ Female	8	0.55	0.33–0.94	0.030	29.33	76.1%	0.000	
	Smoking status								
	Smoker > non-Smoker	13	0.52	0.29–0.96	0.035	52.76	77.3%	0.000	0.129
	Smoker ≤ non-Smoker	9	0.82	0.53–1.27	0.367	31.64	74.7%	0.000	
	Cutoff								
	1% cutoff	3	0.35	0.22–0.55	0.000	1.25	0.0%	0.536	0.440
	5% cutoff	16	0.68	0.43–1.08	0.104	83.17	82.0%	0.000	
	10% cutoff	2	0.54	0.15–1.88	0.332	1.16	13.6%	0.282	
	50% cutoff	5	0.33	0.14–0.81	0.015	2.31	0.0%	0.679	
	Race								
	East-Asian	17	0.68	0.48–0.98	0.037	59.26	73.0%	0.000	0.711
	Other	7	0.45	0.14–1.48	0.188	53.85	75.4%	0.000	
	Primary antibody								
	PoAb^a^	6	0.93	0.46–1.90	0.852	21.55	76.8%	0.001	0.150
	MoAb^b^	17	0.50	0.33–0.75	0.001	61.91	74.2%	0.000	
	Historical type								
	ADC^c^	12	0.69	0.43–1.10	0.119	60.32	81.8%	0.000	0.833
	SCC^d^	2	0.55	0.15–2.02	0.365	1.23	18.4%	0.268	
	Stage								
	I–II	12	0.73	0.45–1.19	0.207	42.67	74.2%	0.000	0.525
	III–IV	2	1.38	0.71–2.69	0.344	0.95	0.0%	0.400	
	Quality								
	M^e^	22	0.67	0.47–0.97	0.033	91.05	76.9%	0.000	0.187
ALK									
	Median age(years)								
	≥60	8	1.74	0.99–3.07	0.056	3.32	0.0%	0.854	/
	<60	2	0.95	0.37–2.43	0.968	1.30	23.2%	0.254	
	Gender								
	Male > Female	6	1.39	0.77–2.49	0.272	1.45	0.0%	0.919	/
	Male ≤ Female	5	1.41	0.74–2.68	0.301	4.69	14.7%	0.321	
	Smoking status								
	Smoker > non-Smoker	5	1.23	0.58–2.57	0.591	2.04	0.0%	0.728	/
	Smoker ≤ non-Smoker	5	1.51	0.84–2.70	0.168	3.82	0.0%	0.432	
	Cutoff								
	5% cutoff	7	1.62	0.99–2.63	0.054	3.45	0.0%	0.750	/
	50% cutoff	2	1.08	0.17–6.96	0.569	0.32	0.0%	0.569	
	Race								
	East-Asian	7	1.52	0.93–2.47	0.095	5.06	0.0%	0.536	/
	Other	4	1.02	0.39–2.66	0.962	0.66	0.0%	0.883	
	Historical type								
	ADC	7	1.30	0.78–2.17	0.138	3.33	0.0%	0.650	/
KRAS									
	Median age (years)								
	≥60	12	1.58	1.23–2.04	0.000	8.51	0.0%	0.667	/
	<60	2	0.71	0.25–2.00	0.517	1.32	24.0%	0.251	
	Gender								
	Male > Female	12	1.34	1.04–1.72	0.025	10.38	0.0%	0.497	/
	Male ≤ Female	4	1.77	1.19–2.62	0.004	1.81	0.0%	0.613	
	Smoking status								
	Smoker > non-Smoker	8	1.35	1.03–1.77	0.032	7.8	10.3%	0.350	/
	Smoker ≤ non-Smoker	6	1.16	0.70–1.19	0.576	4.52	0.0%	0.477	
	Cutoff								
	1% cutoff	2	1.84	1.23–2.74	0.003	0.88	0.0%	0.347	/
	5% cutoff	10	1.35	1.00–1.86	0.049	10.21	11.9%	0.333	
	50% cutoff	5	1.32	0.93–1.89	0.13	0.83	0.0%	0.935	
	Race								
	East-Asian	9	1.24	0.78–1.96	0.358	7.15	0.0%	0.520	/
	Other	7	1.54	1.20–1.96	0.001	5.69	0.0%	0.459	
	Historical type								
	ADC	8	1.69	1.20–2.39	0.003	7.65	8.5%	0.364	/
	SCC	2	0.51	0.09–3.07	0.464	0.26	0.0%	0.609	

Note: a: “PoAb” represents “polyclonal antibody”; b: “McAb” represents “monoclonal antibody”; c: “ADC” represents adenocarcinoma; d: “SCC” represents squamous-cell carcinoma; e: “M” represents moderate quality; 1: p for heterogeneity within each subgroup; 2: p for heterogeneity between subgroups with meta-regression analysis.

### ALK mutation versus ALK wild type

Eleven studies with 3050 clinical specimens were included. No obvious heterogeneity was observed among these studies (*Q* = 6.13, *I*^*2*^ *=* 0.0%, *p =* 0.804). Using a fixed effect model, no statistically significant difference of PD-L1 expression was found among NSCLCs with different ALK status (OR: 1.40, 95% CI, 0.91–2.15, *p =* 0.131) (Figure 2B). On sensitivity analysis, the studies by Koh *et al.* and Zhang *et al.* showed a significant influence on the pooled ORs (Figure 3B). On subgroup analysis, no statistically significant correlation between PD-L1 expression and ALK status was observed (Table 2). The results of Begg’s and Egger’s test (Begg’s *p =* 0.876 and Egger’s *p =* 0.389) (Figure 5C and 5D) indicated a lack of potential publication bias in this respect.

### KRAS mutation versus KRAS wild type

Sixteen studies with 3167 clinical specimens were included. No obvious heterogeneity was found among these studies (*Q* = 13.50, *I*^*2*^ *=* 0.0%, *p =* 0.566). Pooled analysis using a fixed effects model revealed a statistically significant positive correlation between PD-L1 expression and KRAS mutation in NSCLCs (OR: 1.45, 95% CI, 1.18–1.80, *p =* 0.001) (Figure 2C). Respective pooled ORs were stable after sequential elimination of one study at a time (Figure 3C). Statistically significant positive correlation was also found with 1% cutoff, 5% cutoff, non-East-Asian ethnicity, lung adenocarcinoma and elder/smoker dominant groups (Table 2). The results of Begg’s test and Egger’s test showed no existence of potential publication bias (Begg’s *p =* 0.822 and Egger’s *p =* 0.628) (Figure 5E and 5F).

### EGFR/ALK/KRAS mutation versus triple wild type

To further explore the difference in PD-L1 expression between NSCLCs with and without EGFR/ALK/KRAS mutation, we performed a comparison of specimens with at least 1 EGFR/ALK/KRAS mutation and those with triple wild type. Four studies with 840 clinical specimens were included. As compared to triple wild type NSCLCs, those with EGFR/ALK/KRAS mutation did not show any significant differences of PD-L1 expression level with random effect model (OR: 1.51, 95% CI, 0.49–4.71, *p =* 0.475) (Figure 4B). A high level of heterogeneity was found between these studies (*Q* = 31.85, *I*^*2*^ *=* 90.6%, *p =* 0.000).

### Other

Several studies reported the relationship between PD-L1 expression and status of other driver genes, including BRAF, HER2, PIK3CA status and MET expression. We assessed these associations as well. Pooled ORs did not show any significant correlation of BRAF (OR: 1.10, 95% CI, 0.18–6.61, *p =* 0.913), HER2 (OR: 1.00, 95% CI, 0.19–5.22, *p =* 0.996), MET expression (OR: 1.70, 95% CI, 0.63–4.64, *p =* 0.298), and PIK3CA (OR: 1.65, 95% CI, 0.25–11.03, *p =* 0.604) status with PD-L1 expression (Figure 4C).

## DISCUSSION

Clinical efficacy and safety of immune checkpoint inhibitors targeting PD-1/PD-L1 has helped improve prognosis of patients with NSCLCs. There is an urgent need to maximize the clinical benefit of these emerging therapies by optimizing management strategies. The best predictor of the response to PD-1/PD-L1 inhibitors is yet to be determined. A popular hypothesis was that poor treatment outcomes of anti PD-1/PD-L1 therapies may be due to low rates of concurrent CD8+ tumor infiltrating lymphocytes (TILs) and lower PD-L1 expression in the tumor microenvironment (TME) [15].

At cellular level, PD-L1 expression appeared to be associated with strong activation of driver gene (EGFR/ALK) pathways. Up-regulation of PD-L1 level was observed in cell lines with EGFR mutation or ALK rearrangement; further, treatment of EGFR/ALK-TKIs also induced down-regulation of PD-L1 expression [24]. However, the results of preclinical studies are not consistent and have shown lower response rates to anti PD-1/PD-L1 therapies in EGFR/ALK-initiated NSCLCs. The present meta-analysis revealed lower PD-L1 expression in NSCLCs with EGFR mutation. Heterogeneity, which may result from different baseline clinical characteristics of the study population or different investigators for IHC assessment, may influence the accuracy of the conclusions. Besides, we further compared PD-L1 expression levels between exon 19 deletion(del19) and codon 858 mutation in exon 21(L858R), which were the two most common forms of EGFR mutation. Although no significant difference in PD-L1 expression level was found between different EGFR mutated forms owing to limited data, the possibility that heterogeneity resulted from different EGFR mutated forms could not be excluded; additional studies are recommended. In addition, other biological factors like infection may also contribute to heterogeneity [25]. It is reasonable to speculate that lower expression level of PD-L1 in NSCLCs with EGFR mutation may lead to a lower response rate to PD-1/PD-L1 blockade. However, PD-L1 expression level did not differ by EGFR status in the subgroup of patients with 5% cutoff level for PD-L1 positivity, although 5% cutoff has been frequently used in clinical trials. Moreover, the association was found in East-Asian patients but not in patients of other ethnic origin. This result indicates that EGFR mutation may also have potentially influenced the tumor microenvironment. However, no significant correlation between ALK status and PD-L1 positivity was found in this meta-analysis. Studies by Koh *et al.* and Zhang *et al.* obviously influenced the pooled OR. However, pooled ORs were not statistically significant even after elimination of these 2 studies from the analysis.

Prior to introduction of PD1/PD-L1 blockade agents, therapeutic options for NSCLCs with KRAS mutation were extremely limited because the molecular diversity of KRAS-mutant tumors is much greater than that of tumors initiated by other driver genes [26]. The molecular diversity of KRAS-mutant tumors resulted in variable response with the same therapeutic strategy [27]. KRAS mutation was associated with better outcomes of PD1/PD-L1 blockade, which is consistent with the observed correlation of KRAS mutation with higher PD-L1 expression in tumor specimens. Although molecular diversity existed, heterogeneity was not present as expected. The positive correlation was more obvious in the smoker dominant group. Higher levels of PD-L1 expression in tumor samples may explain better outcomes of PD-1/PD-L1 inhibitor therapy in patients with KRAS mutation. Since KRAS mutations are linked to somatic mutational burden, smoking history and expression of neoantigens, our conclusion is in agreement with the emerging concept that tumors with a high mutational burden are more likely to benefit from immunotherapy [28]. Interestingly, driver gene (EGFR/ALK/KRAS) initiated tumors did not show higher levels of PD-L1 as compared to that in triple wild type NSCLCs. This indicated that driver gene (EGFR/ALK/KRAS) mutations may not fully reflect the mutational burden. Furthermore, studies of the relationship of PD-L1 expression and other driver genes like HER2, MET, BRAF and PIK3CA in NSCLCs have been rare. Although none of these genes were found to be correlated with PDL1 expression in our study, the influence of their status on tumor microenvironment deserves further investigation.

This study has a number of limitations. First, whether IHC is an ideal method to assess PD-L1 expression level is debatable. Partial PD 1/PD-L1 blocking response was seen in patient samples who were judged as “PD-L1 negative” by IHC [6, 29]. Second, TME is under dynamic regulation and PD-L1 expression status may alter in different stages of tumors and during treatment. Due to limited data, we could not perform further analysis. Co-mutation of driver gene exists in a certain proportion of NSCLCs, [30]. and we were not able to evaluate whether PD-L1 expression had impact on various co-mutation status. Moreover, the meta-analysis is based on retrospective studies, which may have introduced an element of bias.

## CONCLUSIONS

In conclusion, we found that PD-L1 expression levels in NSCLCs with EGFR mutation were lower than those with EGFR wild type. As compared to the KRAS wild type tumors, those with KRAS mutation tend to express higher levels of PD-L1. ALK, BRAF, HER2, PIK3CA and MET expression status were not significantly associated with PD-L1 expression. As compared to triple negative NSCLCs, driver gene (EGFR/ALK/KRAS) mutations did not alter the level of PD-L1.

## METHOD

### Search strategy

A search for relevant studies was conducted on PubMed, Web of Science and Embase databases. Studies published up to April 26, 2017 were eligible for inclusion. The combinations of key words used are listed in Table 3. Two independent investigators (Bo Lan and Chengxi Ma) were responsible for the assessment of the included studies, and a third investigator (Chengyan Zhang) was involved in resolving any disagreements between the first two investigators.

**Table 3:** Key words used for literature search

#1	(((("Antigens, CD274"[Mesh]) OR B7 H1[Title/Abstract]) OR CD274[Title/Abstract]) OR PD L1[Title/Abstract]) OR Programmed Cell Death 1 Ligand 1 [Title/Abstract]
#2	(("Receptor, Epidermal Growth Factor"[Mesh]) OR Epidermal Growth Factor Receptor) OR EGFR
#3	(((CD246) OR anaplastic lymphoma kinase) OR ALK) OR EML4-ALK fusion
#4	((Kirsten Ras) OR Kirsten rat sarcoma viral oncogene homolog) OR KRAS
#5	((((((((("Lung Neoplasms"[Mesh]) OR Pulmonary Neoplasms[Title/Abstract]) OR Lung Neoplasms[Title/ Abstract]) OR Pulmonary cancer[Title/Abstract]) OR Lung cancer[Title/Abstract])) OR Lung adenocarcinoma [Title/Abstract]) OR Pulmonary adenocarcinoma[Title/Abstract]) OR Lung squamous carcinoma[Title/Abstract]) OR Pulmonary squamous carcinoma[Title/ Abstract]
#6	#2 OR #3 OR #4
	#1 AND #6 AND #5

### Selection criteria

Eligible studies were required to meet the following criteria to be included: 1) Patients were diagnosed with NSCLC by histopathological methods; 2) Expression of PD-L1 in tumor tissues was evaluated by immunohistochemistry and the specific cutoff for PD-L1 positive expression was reported; 3) At least one driver gene was involved in the study; 4) Distribution of driver gene in participants was reported; 5) Sufficient data to form an 2 × 2 table was reported: number of PD-L1 positive specimens with or without driver genes mutation, and number of PD-L1 negative specimens with or without driver genes mutation.

Exclusion criteria were as follows: 1) Studies not related to NSCLC; 2) Studies on patients with metastatic lung cancer; 3) PD-L1 expression was not evaluated in tumor tissues; 4) Other methods were used for evaluation of expression of PD-L1 such as ELISA, gene copy number or RNA expression level; 5) Studies that included patients <18 years of age or those that included patients with more than one cancer; 6) Non-English publication; 7) Overlapping studies; 8) Editorials, letters, reviews, case reports, duplicate publications, and expert opinions.

### Quality assessment

In order to assess the quality of each included study, three investigators (Bo Lan, Shoujie Chai and Pingli Wang) scored the individual studies according to the Strengthening the Reporting of Observational studies in Epidemiology-Molecular Epidemiology (STROBE-ME) statement [31].(supplement). Quality of each included study was appraised on several aspects and each aspect contained several items. We scored the possible values (0, 1) for each item by consensus at a meeting of all involved investigators. Final scores were displayed as percentages of maximal score. According to percentages of final score (Supplementary Table 1), quality of included studies was classified as: “high-quality” (100%–75%); “moderate-quality” (74%–50%); “low-quality” (49%–25%); “very low-quality” (24%–0%).

### Data extraction

Data from the included studies was extracted independently by two investigators (Bo Lan and Chengxi Ma). A third investigator (Chengyan Zhang) was responsible for resolving any disagreement between the first two investigators. The following information were collected: last name of first author, year of publication, ethnicity, median age, gender, stage, smoking status, histological type, primary PD-L1 antibody used for IHC assessment, cutoff level for PD-L1 positive, status of driver genes and PD-L1 expression (Table 1).

### Statistical analysis

All data analysis was performed using software STATA version 14.0 (Stata Corp). Pooled odds ratios (OR) of PD-L1 positive with 95% CI for driver gene status were calculated by fixed or random effect model and forest plots generated. *I*^*2*^ and p statistics were selected as measures of heterogeneity. *P* value < 0.05 or *I*^*2*^ > 0.0% were considered indicative of existence of significant heterogeneity. *I*^*2*^ ≤ 25.0% indicated low heterogeneity while 25% and 75% were defined as the thresholds for moderate and high heterogeneity, respectively. *I^2^* ≤ 50.0% was considered indicative of an acceptable level of heterogeneity [32]. A random effect model was used when significant heterogeneity was found; otherwise, fixed effect model was used. Sensitivity analysis was performed by sequential omission of one study at a time to explore the effect of individual studies on the pooled OR. Subgroup analysis using random effect model was performed to detect association of PD-L1 expression and driver gene status with different population characteristics (median age, cutoff value, ethnicity, histological type, stage, gender and smoking status), as well as to assess the source of heterogeneity. “Cutoff” was defined as the score or percentage of positive staining tumor cells used for classification of a specimen as “PD-L1 positive” in each study. In the event of an unacceptable level of heterogeneity, meta regression with Knapp-Hartung method was used to assess the effect of median age, cutoff level, ethnicity, histological type, stage, gender, smoker and quality of publication. Publication bias was evaluated by Begg’s rank correlation test and Egger’s linear regression test. *P* < 0.05 was considered indicative of statistically significant difference. Once significant publication bias was observed, trim and fill procedure was performed to evaluate the number of missing publications and the pooled ORs with 95% CI adjusted.

## SUPPLEMENTARY MATERIALS TABLES


