# Supplementary material for: Association between PD-L1 expression and driver gene status in non-small-cell lung cancer: a meta-analysis
Source: Oncotarget. 2018 Jan 5;9(7):7684–99. doi: 10.18632/oncotarget.23969 (PMC5800936; doi:10.18632/oncotarget.23969)
Supplement: Supplementary file 1 [file oncotarget-09-7684-s001.pdf]

Association between PD-L1 expression and driver gene status in non-small-cell lung cancer: a meta-analysis

SUPPLEMENTARY MATERIALS

Supplementary Table 1: Results of STROBE quality assessment for cross-sectional studies

Study	Title and abstract	Introduction	Methods	Results	Discussion	Other information	Total score (%)
Zhang	1	2	8	4	3	0	55
Yang	1	2	8	4	3	0	55
Incecoo	1	2	8	4	4	2	64
Cooper	1	2	7	5	4	2	64
Schmidt	1	2	10	5	4	2	73
Kim	1	2	8	3	2	1	52
Yang2	1	2	9	4	3	2	64
Koh	1	2	7	7	4	1	67
Tang	1	2	10	4	2	0	58
Chang	1	2	7	4	2	2	55
Andreas	1	2	10	5	3	2	70
Ameratunga	1	2	8	6	3	2	67
Song	1	2	8	5	3	1	61
Inamura	1	2	8	5	4	2	67
Jia	1	2	9	5	4	2	70
Inoue	1	2	9	7	3	2	73
Ji	1	2	10	4	2	1	61
Huynh	1	2	11	6	4	1	76
Mori	1	2	8	5	4	1	64
Takada	1	2	9	5	2	1	61
Rangachari	1	2	6	5	2	1	46
Tsao	1	2	10	3	3	2	76
Dong	1	2	7	7	4	2	64
Cho	1	2	9	5	3	1	67

Supplementary Table 2: Data pertaining to status of driver genes and PD-L1 expression in each study

Study	PD-L1(+) Diver gene (mutation/WT)	PD-L1(–) Diver gene (mutation/WT)
Zhang	EGFR (37/33), ALK (3/67), KRAS (4/66), Her2 (2/68)	EGFR (39/34), ALK (6/67), KRAS (3/70), Her2 (5/68)
Yang	EGFR (43/22), ALK (2/63), KRAS (5/60), BRAF (4/61), PIK3CA (22/65)	EGFR (54/44), ALK (1/97), KRAS (3/95), BRAF (3/95), PIK3CA (16/177)
Incecoo	EGFR (43/22), ALK (2/63), KRAS (5/60), Triple1 (36/7)	EGFR (16/39), ALK (4/51), KRAS (13/42), Triple (57/22)
Cooper	EGFR (0/15), ALK (0/15), KRAS (7/8)	EGFR (33/222), ALK (3/252), KRAS (81/174)
Schmidt	EGFR (2/8)	EGFR (4/14)
Kim	EGFR (0/57), MET (24/65)	EGFR (7/121), MET (65/177)
Koh	EGFR (128/106), ALK (18/275), KRAS (16/98), MET (117/176)	EGFR (100/65), ALK (5/195), KRAS (9/76), MET (39/164)
Tang	EGFR (64 (L858R 35/ Del19 26)/ 42)	EGFR (25/24)
Omori	EGFR (6/19)	EGFR (23/14)
Chang	EGFR (4/46)	EGFR (4/12)
Yang2	EGFR (9/50), ALK (0/59), KRAS (0/59), BRAF (2/57)	EGFR (9/37), ALK (0/46), KRAS (1/45), BRAF (6/40)
Andreas	KRAS (23/15)	KRAS (32/53)
Ameratunga	5% cutoff: EGFR (7/200), KRAS (43/164); 50% cutoff: EGFR (3/97), KRAS (22/78);	5% cutoff: EGFR (20/300), KRAS (57/363); 50% cutoff: EGFR (24/403), KRAS (78/349);
Song	EGFR (112/74), ALK (10/176), KRAS (5/181), Her2 (6/180), PIK3CA (2/ 184), BRAF (2/184), Triple (161/25)	EGFR (93/106), ALK (8/191), KRAS (11/188), Her2 (3/196), PIK3CA (4/ 195), BRAF (0/199), Triple (131/68)
Inamura	EGFR (4/24), ALK (1/42), KRAS (4/20)	EGFR (89/77), ALK (9/216), KRAS (17/148)
Jia	EGFR (7/9), ALK (1/15), KRAS (1/15)	EGFR (48/44), ALK (4/90), KRAS (8/86)
Inoue	EGFR (25/176), ALK (5/196)	EGFR (107/346), ALK (5/448)
Ji	EGFR (18/22), KRAS (5/35)	EGFR (42/18), KRAS (5/55)
Huynh	EGFR (5/90), ALK (1/94), KRAS (50/45), Triple (56/39)	EGFR (49/117), ALK (3/163), KRAS (58/108), Triple (110/56)
Mori	EGFR (5/90)	EGFR (5/90)
Takada	1% cutoff: EGFR (20/44); 5% cutoff: EGFR (8/32);	1% cutoff: EGFR (92/79); 5% cutoff: EGFR (104/ 91);
Dong	5% cutoff: EGFR (0/7), KRAS (2/5); 50% cutoff: EGFR (0/4), KRAS (1/3);	5% cutoff: EGFR (1/5), KRAS (0/6); 50% cutoff: EGFR (1/8), KRAS (1/8);
Rangachari	EGFR (0/21), ALK (1/20), KRAS (7/14), Triple (8/13)	EGFR (13/37), ALK (3/47), KRAS (16/34), Triple (32/18)
Tsao	1% cutoff: EGFR (5/79), KRAS (44/63); 25% cutoff: EGFR (2/47), KRAS (27/41); 50% cutoff: EGFR (1/32), KRAS (16/26);	1% cutoff: EGFR (30/186), KRAS (83/195); 25% cutoff: EGFR (33/218), KRAS (100/217); 50% cutoff: EGFR (34/233), KRAS (111/232);
Chen	EGFR (16/8)	EGFR (12/16)
Cho	EGFR (64 (L858R 23/ Del19 38))	EGFR (205 (L858R 98/ Del19 107))

Note: 1: Mutation of triple represents “at least 1 gene mutation of EGFR, ALK, KRAS”; Wild type of triple represents “triple wild type of EGFR, ALK and KRAS”.

Supplementary Table 3: Strengthening the Reporting of Observational studies in Epidemiology – Molecular Epidemiology (STROBE-ME)

	Item No	Recommendation
**Title and abstract**	1	State the use of specific biomarker(s) in the title and ⁄ or in the abstract if they contribute substantially to the findings.
**Introduction**		
Background/rationale	2	Explain in the scientific background of the paper how ⁄ why the specific biomarker(s) have been chosen, potentially among many others (e.g. others are studied but reported elsewhere or not studied at all).
Objectives	3	A priori hypothesis: if one or more biomarkers are used as proxy measures, state the a priori hypothesis on the expected values of the biomarker(s).
**Methods**		
Study design	4	Describe the special study designs for molecular epidemiology (in particular, nested case ⁄ control and case ⁄ cohort) and how they were implemented.
Biological sample collection	4.1	Report on the setting of the biological sample collection; amount of sample; nature of collecting procedures; participant conditions; time between sample collection and relevant clinical or physiological endpoints.
Biological sample storage	4.2	Describe sample processing (centrifugation, timing, additives, etc.).
Biological sample processing	4.3	Describe sample storage until biomarker analysis (storage, thawing, manipulation, etc.).
Biomarker biochemical characteristics	4.4	Report the half-life of the biomarker and chemical and physical characteristics (e.g. solubility).
Setting	5	Describe the setting, locations and relevant dates, including periods of recruitment, exposure, follow-up and data collection.
Participants	6	Report any habit, clinical condition, physiological factor or working or living condition that might affect the characteristics or concentrations of the biomarker.
Variables	7	Clearly define all outcomes, exposures, predictors, potential confounders and effect modifiers. Give diagnostic criteria, if applicable
Data source⁄ measurement	8	Laboratory methods: report type of assay used, detection limit, quantity of biological sample used, outliers, timing in the assay procedures (when applicable) and calibration procedures or any standard used.
Bias	9	Describe any efforts to address potential sources of bias
Study size	10	Explain how the study size was arrived at
Quantitative variables	11	Explain how quantitative variables were handled in the analyses. If applicable, describe which groupings were chosen and why
Statistical methods	12	Describe how biomarkers were introduced into statistical models.
Validity ⁄ reliability of measurement and internal ⁄ external validation	12.1	Report on the validity and reliability of measurement of the biomarker(s) coming from the literature and any internal or external validation used in the study.
**Results**		
Participants	13	Give reason for loss of biological samples at each stage.
Descriptive data	14	(a) Give characteristics of study participants (e.g. demographic, clinical and social) and information on exposures and potential confounders.
		(b) Indicate the number of participants with missing data for each variable of interest.
		(c) Cohort study – Summarize follow-up time (e.g. average and total amount).
Distribution of biomarker measurement.	14.1	Give the distribution of the biomarker measurement (including mean, median, range and variance).
Outcome data	15	Cohort study – Report numbers of outcome events or summary measures over time. Case–control study – Report numbers in each exposure category or summary measures of exposure. Cross-sectional study – Report numbers of outcome events or summary measures.
Main results	16	(a) Give unadjusted estimates, and, if applicable, confounder-adjusted estimates and their precision (eg, 95% confidence interval). Make clear which confounders were adjusted for and why they were included.
		(b) Report category boundaries when continuous variables were categorized
		(c) If relevant, consider translating estimates of relative risk into absolute risk for a meaningful time period
Other analyses	17	Report other analyses done, e.g., analyses of subgroups and interactions, and sensitivity analyses
**Discussion**		
Key results	18	Summarize key results with reference to study objectives
Limitations	19	Describe main limitations in laboratory procedures.
Interpretation	20	Give an interpretation of results in terms of a priori biological plausibility.
Generalizability	21	Discuss the generalizability (external validity) of the study results.
**Other information**		
Funding	22	Give the source of funding and the role of the funders for the present study and, if applicable, for the original study on which the present article is based.
Ethics	22.1	Describe informed consent and approval from ethical committee(s). Specify whether samples were anonymous, anonymized or identifiable.
